# Feature-guided deep learning reduces signal loss and increases lesion CNR in diffusion-weighted imaging of the liver

**DOI:** 10.1016/j.zemedi.2023.07.005

**Published:** 2023-08-04

**Authors:** Tobit Führes, Marc Saake, Jennifer Lorenz, Hannes Seuss, Sebastian Bickelhaupt, Michael Uder, Frederik Bernd Laun

**Affiliations:** a Institute of Radiology, University Hospital Erlangen, Friedrich-Alexander-Universität Erlangen-Nürnberg (FAU), Erlangen, Germany; b Department of Radiology, Klinikum Forchheim – Fränkische Schweiz, Forchheim, Germany

**Keywords:** Deep learning, Neural network, Liver, Diffusion, Cardiac motion artifact, Oncology

## Abstract

****Purpose**:**

This research aims to develop a feature-guided deep learning approach and compare it with an optimized conventional post-processing algorithm in order to enhance the image quality of diffusion-weighted liver images and, in particular, to reduce the pulsation-induced signal loss occurring predominantly in the left liver lobe.

****Methods**:**

Data from 40 patients with liver lesions were used. For the conventional approach, the best-suited out of five examined algorithms was chosen. For the deep learning approach, a U-Net was trained. Instead of learning “gold-standard” target images, the network was trained to optimize four image features (lesion CNR, vessel darkness, data consistency, and pulsation artifact reduction), which could be assessed quantitatively using manually drawn ROIs. A quality score was calculated from these four features. As an additional quality assessment, three radiologists rated different features of the resulting images.

****Results**:**

The conventional approach could substantially increase the lesion CNR and reduce the pulsation-induced signal loss. However, the vessel darkness was reduced. The deep learning approach increased the lesion CNR and reduced the signal loss to a slightly lower extent, but it could additionally increase the vessel darkness. According to the image quality score, the quality of the deep-learning images was higher than that of the images obtained using the conventional approach. The radiologist ratings were mostly consistent with the quantitative scores, but the overall quality ratings differed among the readers.

****Conclusion**:**

Unlike the conventional algorithm, the deep-learning algorithm increased the vessel darkness. Therefore, it may be a viable alternative to conventional algorithms.

## 1 Introduction

Detection or rule-out of liver metastases is an important clinical application of MRI. Many cancer types tend to metastasize to the liver, making metastases about 18–40 times more common than primary cancer in the liver [1], [2]. As many malignancies are not diagnosed at an early stage and symptoms often first appear after liver metastases have occurred [3], [4], the reliable detection of metastases has a considerable impact on patient outcomes.

The use of magnetic resonance imaging and, in particular, diffusion-weighted imaging (DWI), is pivotal in the diagnosis of liver cancer and liver metastases [5], [6], [7], [8], [9], [10]. Unfortunately, DWI of the liver is prone to artifacts due to the close proximity of the heart [11], [12], [13], [14], [15], resulting in signal loss, mainly in the left lobe of the liver, and the potential disappearance of lesions in this region [16]. Several approaches exist to mitigate this artifact: the use of electrocardiography (ECG) triggering [17], [18], flow-compensated diffusion gradient waveforms [12], [19], [20], [21], [22], [23], [24], [25], [26], [27], or post-processing techniques [28], [29], [30], [31].

Most post-processing techniques work according to the same principle: give higher weight to bright pixels during averaging and thus make those regions brighter, where the intensity differs strongly among the repetitions. This approach is well suited for regions affected by signal dropouts at some repetitions, such as those caused by cardiac pulsation. However, this procedure can also have undesirable effects on regions that sometimes experience random increases in brightness, such as vessels. Their brightness in the post-processed image could also increase, with the risk that they could be mistaken for lesions. This means that care must be taken to increase the brightness of dark areas sufficiently without increasing vessel brightness too much.

Accordingly, it has recently been shown that it is useful to monitor this trade-off by assessing the quality of the post-processed images quantitatively. Four image features have been proposed to reflect these issues [32]: the severity of the signal voids due to cardiac pulsation, the retention of dark vessels, the contrast-to-noise ratio of lesions, and data consistency in areas that are not affected by artifacts.

Apart from “conventional” post-processing techniques, it is not surprising that deep learning-based methods [33], [34] are used with great success to improve MR images in various tasks, such as correcting breathing artifacts in liver DCE imaging [35], the correction of rigid head motion [36], or EPI distortion correction [37], just to name a few.

Therefore, it seems reasonable that deep learning can also improve images that are corrupted by artifacts due to cardiac pulsation.

The standard approach would be to train a network with pairs of corrupted and non-corrupted images. This approach comes with two drawbacks. First, it can be difficult to obtain truly uncorrupted images since the pulsation artifact is common among the unaveraged images and the blood signal is volatile [12]. Second, this approach seems inadequate for actually enhancing image quality beyond the level of the uncorrupted images. Therefore, we decided to use a “feature-guided” approach: Given the proven usefulness of the above features to assess image quality, we decided to train the network such that it optimizes these four features.

We then compared the results to the optimized “conventional” algorithms (as proposed in [32]) to evaluate whether deep learning performs better or not.

## 2 Methods

### 2.1 Imaging

We used a data set of 40 patients with liver lesions, which had previously been acquired in an IRB-approved prospective study [26]. All patients gave informed consent prior to the examinations for acquiring and evaluating the data, which includes the evaluation performed here. For each patient, 39 slices had been acquired with a single-refocused diffusion spin-echo echo-planar imaging research application sequence with free breathing on a MAGNETOM Aera (Siemens Healthcare, Erlangen, Germany) at a field strength of 1.5 T. The acquisition parameters are shown in Table 1.

**Table 1:** Sequence parameters.

TE	70 ms
TR	12,400 ms
Bandwidth	2790 Hz/px
Echo spacing	0.49 ms
Phase partial Fourier factor	6/8
Diffusion weightings (repetitions)	50 s/mm^2^ (1x3), 800 s/mm^2^ (4x3)
Diffusion directions	(1, –1, 0.5)^T^, (–1, –0.5, 1)^T^, (–0.5, –1, –1)^T^
FoV	400 mm × 325 mm
Matrix size	128 × 104 (interpolated to 256 × 208)
Slice thickness	5 mm
Slice distance	1 mm
Fat suppression	SPAIR
Parallel acquisition	GRAPPA (acceleration factor: 2, 24 reference lines)

### 2.2 Quantitative image quality assessment

For the quantitative assessment of the image quality, we use a method developed by Führes et al. [32]. It relates the image quality of a post-processed image to the quality of the unprocessed image by evaluating four image features:

• the severity of the pulsation artifact (PA).
• the vessel darkness (VD).
• the contrast-to-noise ratio of the lesions against the background (CNR).
• the data consistency in regions that are not corrupted by artifacts (DC).

These values can be obtained by segmenting different image regions and calculating the brightness in each region. For example, the value for the PA feature is calculated as the ratio of the mean intensity in the left liver lobe to the mean intensity in the right liver lobe. A more detailed description of the features is provided in the Supporting Information. All four values are designed in such a way that a better feature representation results in a higher value.

These four values were calculated for both the unprocessed trace-weighted image (the “reference image”) and the post-processed image and normalized to the value of the reference image (i.e., the value of the reference image is 1, and the difference of the other value from 1 indicates how much this feature changes). The average of the four scores is then referred to as the total quality score $Q_{total}$. A value larger than 1 indicates an increase in image quality compared with the trace-weighted reference image. More details can be found in the Appendix B and in [32].

The segmentations required for this procedure were created in a total of 198 slices.

### 2.3 Conventional algorithms

In the following, “conventional” refers to algorithms that are not based on deep learning.

Five conventional algorithms were considered:

• Weighted averaging algorithm (inspired by Ichikawa et al. [29])
• Outlier exclusion algorithm (inspired by “informed RESTORE” by Chang et al. [31])
• P-mean algorithm (by Liau et al. [28])
• Percentile algorithm
• Exception set algorithm (by Arning et al. [38])

The algorithms are described in more detail in the Supporting Information.

These algorithms can be run with different parameter settings. For each algorithm and parameter setting, the quality score $Q_{total}$ was calculated (same approach as performed for another data set in Führes et al. [32], but with different data here, i.e. not flow-compensated). The algorithm-parameter combination with the highest $Q_{total}$ value was chosen for comparison with the deep learning approach.

### 2.4 Deep Learning

#### 2.4.1 Network architecture

**Figure 1:**
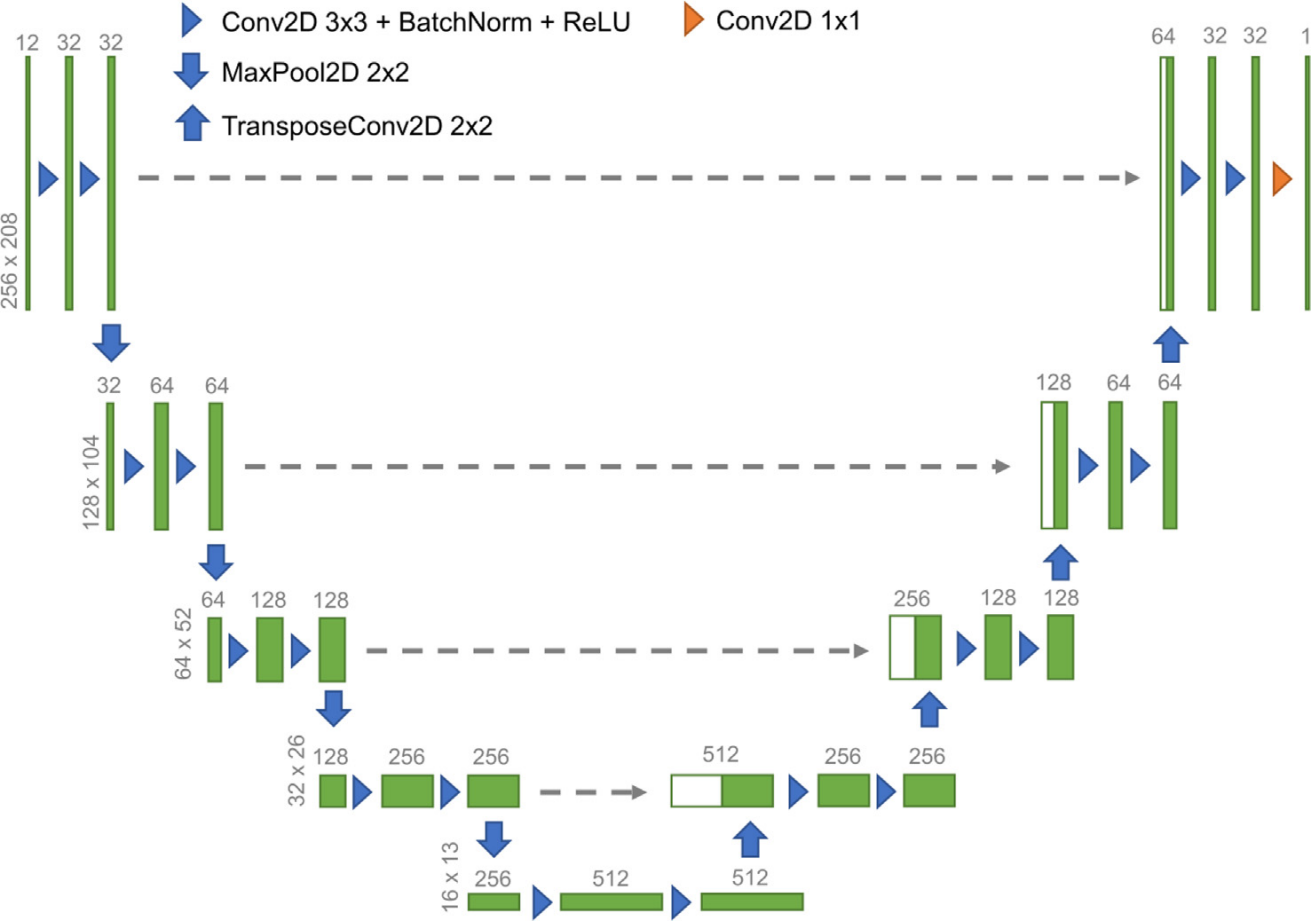
U-Net architecture. The green boxes represent input images and feature maps. The white boxes in the right part are copied versions of the boxes in the left part. Upright numbers denote the width and height of the images/feature maps; horizontal numbers denote the channels.

We chose a U-Net [39] architecture for our network (c.f. Fig. 1). For the training, we used the Adam optimizer [40] (learning rate = 0.0001, $\beta_{1}=0.9,\beta_{2}=0.999$). The network was implemented using the PyTorch framework [41] (version 1.12.1).

#### 2.4.2 Loss function

A slightly adapted version of the above-mentioned “total quality score” was used as the loss function L. The adaptation was necessary to prevent the network from learning only one feature.

$$L=-\lambda_{DC}DC-\lambda_{CNR}CNR-\lambda_{PA}PA-\lambda_{VD}VD+\lambda_{DI}DI+\lambda_{\mathrm{PA},2}{\mathrm{PA}}_{2}+\lambda_{\xi }\xi$$

DC, CNR, PA, and VD are defined as specified above. The minus signs are necessary because some features have higher values for increased image quality, and the optimizer aims to minimize the loss function.

DI stands for the dispersion of these four parameters and is defined as follows:

DI=Var(DC,CNR,PA,VD).

The dispersion is used to penalize an outlier of these parameters, which can occur if one of the four scores diverges. In the preliminary evaluations, we observed this for the CNR subscore, whose denominator can become small if the network produces a constant signal intensity over the entire liver. The DI score ensures that an optimal value of the loss function can only be achieved when the values for DC,CNR,PA, and VD are similar.

$PA_{2}$ is an additional measure for reduction of pulsation artifacts. For this, a maximum intensity projection is performed along the dimension of the repetitions. That is, if the image obtained in repetition n is $I_{n}\left(x,y\right)$, then

$$MIP\left(x,y\right)=\max_{n}I_{n}\left(x,y\right)$$

is computed for each voxel. Then this MIP image is compared with the image predicted by the network, $I_{pred}\left(x,y\right)$:

$${\mathrm{PA}}_{2}=\langle MIP\left(x,y\right)\;|\left(x,y\right)\in R_{1}\rangle -\langle I_{pred}\left(x,y\right)|\left(x,y\right)\in R_{1}\rangle .$$

Here, 〈·〉 denotes the average over the selected voxels. $R_{1}$ is the segmented region in the left liver lobe. The ${\mathrm{PA}}_{2}$ score is used to make the brightness increase in the left liver lobe more favorable than the darkening of the right liver lobe. It thus corrected a shortcoming of the above-mentioned PA score, which we observed in preliminary evaluations. Specifically, the PA score only rates the signal ratio between the left and right lobe and thus the network tended to decrease the signal in the right liver lobe. We mitigated this problem by introducing ${\mathrm{PA}}_{2}$ to favor a brightening of the left liver lobe over darkening of the right liver lobe.

ξ denotes the $L_{2}$ norm between reference image $I_{ref}\left(x,y\right)$ and predicted image:

$$\xi ={\|I_{pred}\left(x,y\right)-I_{ref}\left(x,y\right)\|}_{2}$$

This term is used to preserve the overall image impression and to keep the image background dark.

The loss was calculated on a larger set of segmentations (328 slices) than for the conventional algorithm in order to have more data to train on.

The weights were set as follows: $\lambda_{\mathrm{DC}}=0.5$, $\lambda_{\mathrm{CNR}}=1.5$, $\lambda_{\mathrm{PA}}=1$, $\lambda_{\mathrm{VD}}=1$, $\lambda_{\mathrm{DI}}=0.5$, $\lambda_{\mathrm{PA},2}=0.05$, $\lambda_{\xi }=0.05$. In preliminary experiments, we attempted to use equal weights $\lambda_{\mathrm{DC}},\lambda_{\mathrm{CNR}},\lambda_{\mathrm{PA}},$ and $\lambda_{\mathrm{VD}}$, but we observed that a higher value was necessary for $\lambda_{\mathrm{CNR}}$ to obtain a sufficient CNR. A lower value was necessary for $\lambda_{\mathrm{DC}}$ to allow for stronger image processing (as DC quantifies the deviation in the right liver lobe). The weight $\lambda_{\mathrm{DI}}$ was set just as high as necessary to avoid a too large dispersion of the scores (such as the CNR, see above). The values for $\lambda_{\mathrm{PA},2}$ and $\lambda_{\xi }$ were set to 0.05 to force their respective contributions to the loss function to be approximately the same order of magnitude as the other contributions.

#### 2.4.3 Dataset preparation

The twelve images per slice were normalized (to a zero mean and a standard deviation of 1). They were randomly translated in left-right and bottom-top directions by up to 10% of their width or height, respectively (all twelve by the same distance). They were also randomly scaled by a factor between 90% and 105%. The twelve images were then provided as input for the network. The data were split into a training set, containing 80% of the slices, and a test set containing 20% of the slices. Data were split patient-wise, which means that all slices from one patient were either in the test set or the training set.

#### 2.4.4 Training

Training was performed on an Nvidia GeForce RTX 3090 GPU. The batch size was 64.

Before starting the training, the network weights were pretrained on the trace-weighted reference images for 3000 epochs.

The actual training was then performed until the average loss over the latest 100 epochs was not lower than for the 100 epochs before that. As a final result, the weights that achieved the lowest loss were used.

### 2.5 Image quality assessment by readers

Three board-certified radiologists assessed the image quality of the post-processed images in a blinded, randomized evaluation. For each of the seven patients in the test set, the radiologists compared all the slices from both the conventional and the deep learning approach with the unprocessed slices. They gave six scores per patient, one in each of the following categories:

• Signal in the left liver lobe
• Darkness of vessels
• Lesion CNR
• Perceived image resolution
• Perceived image noise
• Overall image quality

For both the conventional and deep learning approach, they rated each category with a score from −3 to 3, where 0 referred to “as good as the unprocessed image”. The meanings of the other scores are shown in Table 2. For the evaluation, all the scores were averaged in each category.

**Table 2:** Meaning of the different scores for the reading by radiologists.

Value	Meaning
-3	Much worse than the unprocessed image
-2	Worse than the unprocessed image
-1	Slightly worse than the unprocessed image
0	as good as the unprocessed image
1	Slightly better than the unprocessed image
2	Better than the unprocessed image
3	Much better than the unprocessed image

## 3 Results

### 3.1 Conventional algorithm

**Figure 2:**
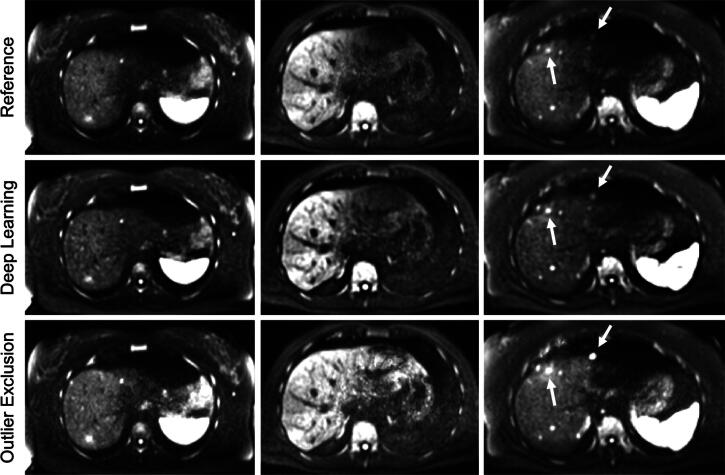
Comparison of the reference image, the result of the deep learning algorithm, and the result of the outlier exclusion algorithm. In the first column, both algorithms reduce the signal loss in the left lobe. In the second column, the left lobe is brighter in the outlier exclusion image, but structures in the left lobe and vessels are better identifiable in the deep learning image. In the third column, the lesion signal is highest for outlier exclusion (left and right arrow), but the contrast is better for the deep learning algorithm (left arrow).

The algorithm with the highest value of $Q_{\text{total}}$ was the outlier exclusion algorithm with the following parameters: number of exclusion iterations k=10, kernel size of the low-pass filter for the standard deviation map $ks_{2}=21$, and the correction threshold thr=0.3. The algorithm and the exact meaning of these parameters are described in the Appendix A. Moreover, the detailed optimization and performance report for all five algorithms is shown in the Supporting Information. $Q_{\text{total}}$ was 1.115. The values for the feature scores can be seen in Table 3. The pulsation artifact could be substantially reduced and the CNR of the lesion increased. Vessel darkness and data consistency are reduced. Fig. 2 shows representative images from three patients. The left liver lobe appears brighter in the outlier exclusion images in all cases. One lesion marked with a white arrow is better visible in the outlier exclusion images; the other becomes brighter but the contrast decreases.

**Table 3:** Values of image feature scores. Values larger than one are printed in bold. PA = severeness of pulsation artifact, DC = data consistency, VD = vessel darkness, CNR = contrast-to-noise ratio, $Q_{total}=$ total quality score.

	PA	DC	VD	CNR	$Q_{\text{total}}$
Conventional	**1.556**	0.862	0.799	**1.241**	**1.115**
Deep Learning	**1.322**	0.862	**1.268**	**1.172**	**1.156**

### 3.2 Deep Learning

The training was stopped after 284 epochs because the test loss did not decrease any more. $Q_{\text{total}}$ in the test data was 1.156. The values for the feature scores are shown in Table 3. The deep learning algorithm improved the scores of the pulsation artifact, the lesion CNR, and the vessel darkness compared to the reference image. Only the data consistency score decreased. Fig. 2 shows representative images. Structures in the left liver lobe and vessels are better identifiable than in the reference image.

### 3.3 Image quality comparison

#### 3.3.1 Comparison by scores

The scores for the different image features show that both algorithms perform rather similarly in reducing pulsation artifacts, increasing the lesion CNR, and maintaining the data consistency, with the conventional algorithm being slightly better. For the vessel darkness, the deep learning approach performs much better: it even increases the darkness of the vessels, while the conventional algorithm is unable to maintain the vessel darkness.

Overall, the conventional algorithm improved two of the four image features, and the deep learning approach three of the four features. This is the key reason why the total quality score is higher for the deep learning images.

#### 3.3.2 Visual comparison

**Figure 3:**
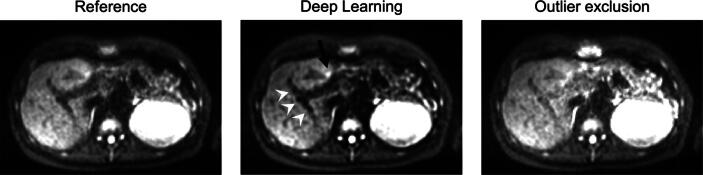
A representative slice for which deep learning performs better than the outlier exclusion algorithm. The white arrowheads in the middle image show a vessel that is much better identifiable in the deep learning image. The black arrow shows a lesion whose CNR is strongly increased.

**Figure 4:**
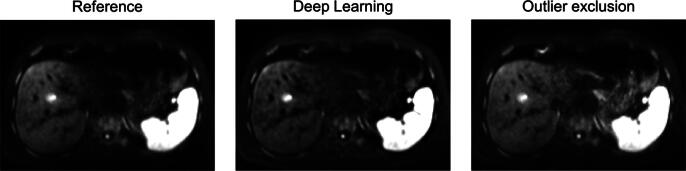
A representative image demonstrating the sometimes-occurring different visual appearance of the left liver lobe.

Comparisons between the results of the conventional and the deep learning approach are additionally shown in Figure 2, Figure 3, Figure 4. The left liver lobe appears brighter in all cases compared to the reference image. The ventral lesion marked by a white arrow in Fig. 2 is better visible in the outlier exclusion images; the more central lesion becomes brighter but the contrast decreases. In general, the liver tissue appears slightly brighter with the conventional algorithm and slightly darker in the deep learning images. The deep learning images appear slightly coarse-grained, while the noise in the conventional images tends to be high-frequent. Fig. 3 shows an example where the visibility of a lesion near major vessels was increased in the deep learning image compared to the image obtained with the conventional algorithm. This image, which contains large vessels, appears to be better corrected by deep learning as it recognizes the vessels as vessels and does not attempt to increase their brightness (white arrow heads). Fig. 4 shows an example demonstrating the different visual appearances of the left liver lobe that sometimes occur. The left liver lobe appears brighter with the conventional algorithm, but the deep learning image is more homogeneous in this region. Even though the deep learning image is a bit darker, it looks less noisy.

#### 3.3.3 Comparison by readers

The results of the reading are shown in Table 4. All three readers found that the signal in the left liver lobe improved using both approaches. They all scored the conventional algorithm higher in this category. The same holds true for the lesion CNR, except one reader rated deep learning higher. They also found that the vessel darkness became worse for the outlier exclusion algorithm and better for the deep learning approach. One of the three readers did not observe differences in either the resolution or noise. The other readers rated the resolution for the conventional algorithm higher but rated the noise worse than for the deep learning algorithm. All readers rated the overall image quality of both the conventional and deep learning algorithm higher than that of the unprocessed image. Reader 1 rated the image quality of the two algorithms similarly, Reader 2 rated the conventional algorithm higher, and Reader 3 rated deep learning higher.

**Table 4:** Scores (mean and standard deviation) from radiologist reading. DL = deep learning.

	Signal left lobe		Vessel darkness		Lesion CNR		Resolution		Noise		Overall image quality	
	Conv	DL	Conv	DL	Conv	DL	Conv	DL	Conv	DL	Conv	DL
Reader 1	1.86±0.64	1.14±0.64	-0.43±0.73	1.29±0.70	1.29±0.88	1.14±0.35	0	0	0	0	1.29±1.16	1.14±0.64
Reader 2	2.00±0.53	0.29±0.70	-0.43±0.49	0.43±0.49	0.57±0.73	0.14±0.35	-0.29±0.70	-0.71±0.45	-0.71±1.03	0.57±0.49	1.57±1.17	0.71±0.70
Reader 3	2.14±0.83	0.86±1.46	-1.43±0.73	0.86±1.25	0.43±0.49	1.14±0.83	0.29±0.70	-0.57±0.90	-0.86±0.64	-0.43±0.90	0.57±0.90	1.00±0.76

## 4 Discussion

In this work, we evaluated how well feature-guided deep learning performs to enhance the image quality of artifact-corrupted diffusion data of the liver. We additionally compared it to the performance of a conventional algorithm selected from five possible candidates.

The outlier exclusion algorithm brightens dark areas very effectively. This is a great advantage for the correction of pulsation-induced signal voids in the left liver lobe. Voxels of repetitions that suffer from signal loss are reliably excluded from the calculation of the final image. This leads to a large increase in brightness; however, it can also lead to increased noise as fewer voxels are averaged, but this was not a major problem in our images. Conversely, brightening dark areas can also be a disadvantage in vessel regions. The outlier exclusion algorithm was designed such that it can compensate for an accidentally high signal in one repetition. However, if vessels appear bright in at least two of the twelve repetitions, then the outlier exclusion algorithm will recognize the dark images as outliers and iteratively exclude them. This is a particular problem for large vessels as the algorithm only corrects larger regions where the variation between the repetitions is greater. Small vessels are blurred by the low-pass filter and therefore not affected as strongly. The algorithm performs quite well regarding the increase of the lesion CNR, especially in the left liver lobe.

The deep learning approach does not just focus on brightening dark areas, as its functionality is learned during training and not pre-set. One problem with the algorithm is that the pulsation artifact feature score (PA) is defined as the ratio between the mean brightness of the left and right liver lobes. This means that the score also gets higher (and therefore more favorable) if the left liver lobe remains dark and the right liver lobe is also darkened. Therefore, we introduced the second term in the loss function, PA_2_, which increases the loss if the left liver lobe is darker than it could be with the brightest available voxels. This measure worked well, but it could not completely avoid a slight darkening of the right lobe. One main positive aspect of the network is that it can identify vessels and prevent them from being brightened. This solves the main problem of the conventional algorithm, as bright vessels always bear the risk of being mistaken for lesions. The fact that it even makes them darker may also be viewed positively because it increases image contrast. Fig. 3 shows that even lesions in close proximity to vessels are detected as such and brightened, whereas the vessel is darkened. In general, the respective score indicates that lesion CNR is not increased as strongly as with the conventional algorithm, but mostly still to an extent that allows reliable detection (as seen in the representative image examples). The presented images give the impression that lesion detection is more trustworthy with deep learning than with the conventional algorithm due to its different capabilities to detect vessels. However, this may not hold true in all cases; the risk that faint spots in the images are brightened even if they are not lesions must always be taken into consideration.

Although the quality score $Q_{\text{total}}$ indicated that the image quality was higher for the deep learning approach, the readings of the radiologists suggested that it is difficult to make a clear statement about the quality. This is an interesting finding because, in those features that were part of both evaluations (signal in the left lobe, lesion CNR, vessel darkness), the results were similar to those of our evaluation. Even the two additional properties (image noise and resolution) did not show a strong trend in one direction. This means that for the overall image quality, each of the readers either took also other properties into account (like whether the image looks “common”) or individually weighted some properties stronger than others. As radiologists are trained to detect and characterize lesions, it is possible that some of them prefer higher lesion CNR and more signal in the left liver lobe, and others may have a higher appreciation for reduced vessel brightness, because this also contributes to more reliable lesion detection. The differences among the readers emphasizes the difficulty in clearly defining image quality. This supports our approach of using an automated quality evaluation, instead of allowing the quality to be rated subjectively by radiologists with different individual backgrounds. In terms of our evaluation, different individual backgrounds correspond to various weightings for the different categories. Notably, the variation in weightings can significantly affect the outcome.

In general, the task of making the network learn all features simultaneously and equitably is not easy. The reason is that some scores could diverge easily if the normal $Q_{\text{total}}$ score was used as a loss function. For example, it is possible to maximize the CNR by setting the noise close to zero. This results in an image that is isointense everywhere, and only the lesions appear as bright spots. The CNR feature score used here would then strongly increase, but the other scores would maximally decrease from one to zero. The $Q_{\text{total}}$ score as the mean of the feature scores is then still high and indicates good image quality, even if the image impression has completely changed. Therefore, it was important to incorporate some additional factors into the loss function: The inclusion of variance in the feature scores prevents one score from increasing or decreasing inappropriately strong. Then, a minimum of the loss function can only be found if the values are not too different. Moreover, the L2 norm between the predicted image and the reference image prevented the image from appearing completely different. However, the weight of this factor has been set small enough to not let the network learn just the reference image. Thus, it only prevented strong deviations that are not assessed by other factors like a brightening of the image background. This modified total quality score, which includes the additional factors, could be used for comparison. However, the changes were designed to optimize the network training, rather than to indicate a high image quality. For example, low variance in the feature scores does not directly imply high image quality. Moreover, we wanted to maintain the consistency of our results with those presented in [32].

However, the observation that the network tends to focus on certain features may be beneficial depending on the application. For example, an image with bright lesions and a dark, homogeneous background can be used for automatic lesion segmentation. However, this was not the aim of this work and would require a more detailed examination.

Tamada et al. [42] also examined a deep learning approach by training a classifier to detect artifact-corrupted repetitions and only averaging non-corrupted repetitions. The results were promising to a certain extent, but it does not seem efficient to exclude complete repetitions if the artifact is only restricted to a certain region. Deep-learning guided patch-wise weighted repetition averaging was used by Gadjimuradov et al. to improve on this method [30]. They used quadratic patches of images and determined a pixel-wise weight for each repetition by comparing the pixel intensity to the medians of the mean and standard deviation of the repetition patches. They used the deep learning classifier to detect repetitions corrupted by signal voids and excluded them from the calculation of the median. They report successful correction of signal dropouts in the liver with better results than Tamada et al. However, they mainly tested their method on volunteer data and applied it only to one patient, so the significance in this regard remains limited.

The concept of a feature-guided deep learning approach seems to work quite well. A key aspect is that the desired result is not known a priori. Normally, U-Nets are used to map an input image as accurately as possible onto the target image. This bears the disadvantage that the result can hardly be better than the target images. It means that the result strongly depends on the quality of the available target images. Here, we use the network to find the image that represents the image features best. Even if some additional conditions are needed in the loss function to “put it on the right track”, the network increases the image quality without ever having seen a clean, uncorrupted image. Of course, this result also depends on the accuracy of the segmented regions.

In addition to postprocessing techniques, the signal loss can be reduced during the acquisition by employing flow-compensated or first moment-nulled gradient waveforms. Normally, constantly moving spins accumulate a phase that leads to signal loss when different velocities are present [19], [21], [23]. With flow-compensated waveforms, this phase accumulation is compensated, which partly reduces the signal loss. Several recent technical advances include the generation of waveforms with optimized echo time [43] and with compensation for effects caused by concomitant fields [44]. However, the drawback is that the blood vessels generally appear as bright spots. Thus, as observed in the present work, there is a trade-off between signal dropout correction and retention of dark vessels. For the gradient waveform approach, a possible solution is the use of small flow-weightings, as proposed by Zhang et al. [45]. For post-processing, our proposed deep learning approach is a possible solution. A combination of both methods may be worth testing.

We also have to acknowledge some limitations of this study. Even though we augmented the training data by shifting and scaling, more data could have yielded more reliable results. Additionally, it might have been useful to use the other images from the clinical protocol (e.g., T2-weighted images) as input information, but this would have required image registration and would have led to increased training duration. Moreover, there is no agreement on a standard method for assessing image quality in liver DWI. This was highlighted by the divergent ratings we received from the board-certified radiologists. Therefore, we used our developed method, even though it does not include all the potentially relevant image features. In addition, the choice of weightings λ in the loss function influences the results. The performance might change for a different choice or a different data set. It is important to consider that this choice is not necessarily suited for every possible data set and some changes might be required. Since we only used data from only one scanner with one set of acquisition parameters, the generalizability of this approach might also be limited, which has not been investigated herein.

### 4.1 Conclusion

In conclusion, the presented feature-guided deep learning approach is suitable to improve the image quality of corrupted liver DWI images. It can enhance three of the four examined image features. The main difference compared with the conventional algorithm is that, even though this performs slightly better in three of the four feature scores, it does not brighten vessels in the image. Therefore, the deep learning approach is a viable alternative to the conventional algorithm. Application of the deep learning approach to other regions affected by signal loss, such as the heart [46], [47] or the muscle [48], appears promising.

## Declaration of Competing Interest

The authors declare that they have no known competing financial interests or personal relationships that could have appeared to influence the work reported in this paper.

## Figures and Tables

**Figure A1:**
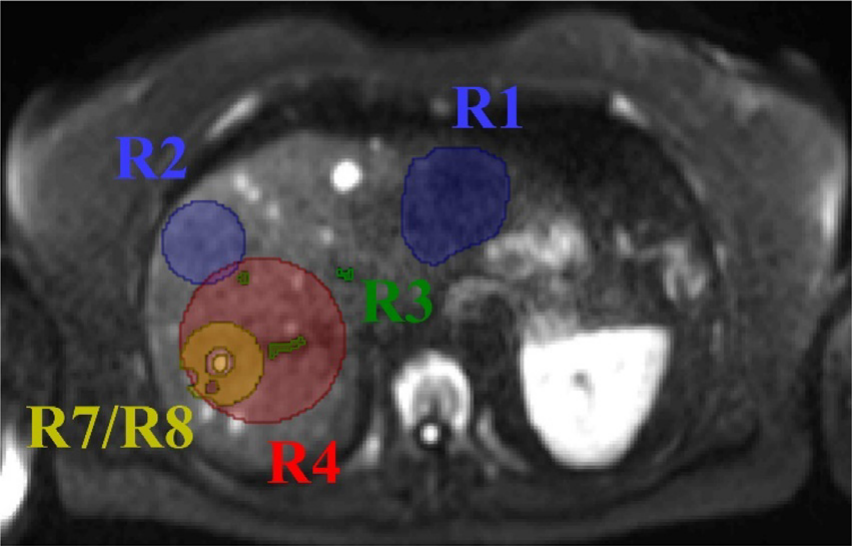
Segmented image regions

**Figure A2:**
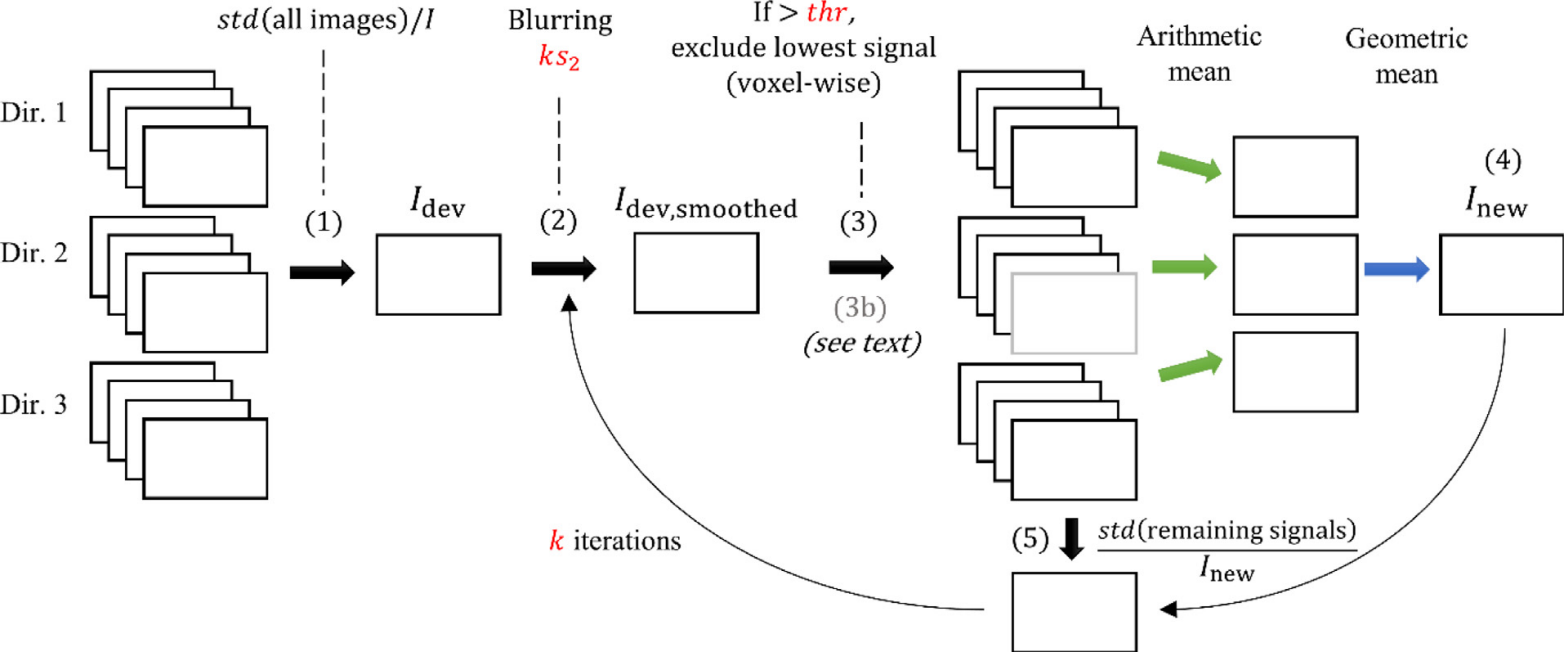
Outlier exclusion algorithm. Figure similarly published in [32].

## Appendix A

### 1 Image features

For the assessment of the image quality, the following regions were segmented: See Fig. A1

R1: Region in the left liver lobe affected by signal loss

R2: Region (10 cm^2^) in the right liver lobe not affected by signal loss

R3: Vessels

R4: Region (40 cm^2^) in the right liver lobe

R5/R7: Lesion in the left/right lobe

R6/R8: Tissue surrounding the lesion in the left/right lobe

Four image features are considered:

In the following, I(x,y) denotes the evaluated image (one slice), and $I_{ref}\left(x,y\right)$ denotes the reference image (the same slice). 〈·〉 denotes the average over all included voxels.

Pulsation artifact feature PA:

$$\mathrm{PA}=\frac{\langle I\left(x,y\right)|\left(x,y\right)\in R1\rangle }{\langle I\left(x,y\right)|\left(x,y\right)\in R2\rangle }$$

Vessel darkness feature VD:

$$\mathrm{VD}=\frac{\langle I\left(x,y\right)|\left(x,y\right)\in R2\rangle -\langle I\left(x,y\right)|\left(x,y\right)\in R3\rangle }{\langle I\left(x,y\right)|\left(x,y\right)\in R2\rangle }$$

Data consistency feature DC:

$$\mathrm{DC}=1-\langle \frac{I\left(x,y\right)-I_{ref}\left(x,y\right)}{I_{ref}\left(x,y\right)}|\left(x,y\right)\in R4\rangle$$

Contrast-to-noise ratio CNR:

$$\mathrm{CNR}=\frac{\langle I\left(x,y\right)|\left(x,y\right)\in R7\rangle -\langle I\left(x,y\right)|\left(x,y\right)\in R8\rangle }{\mathrm{std}(I\left(x,y\right)|\left(x,y\right)\in R8)}$$

The scores were normalized to the scores of the respective reference image. Afterwards, they were averaged over all slices (in the case of deep learning, all slices of the batch).

The mean of the averaged scores was used as total quality score $Q_{total}$.

### 2 **Outlier exclusion algorithm**

The algorithm iteratively excludes the lowest signal per voxel in regions where the signal deviation between the repetitions is higher than a threshold value. See Fig. A2.

In the following, $I^{n}\left(x,y\right)$ denotes the i-th repetition, and $I_{ref}\left(x,y\right)$ denotes the trace-weighted image.

(1) a relative standard deviation map $I_{dev}\left(x,y\right)$ is calculated:$I_{dev}\left(x,y\right)=\frac{\mathrm{std}\left(I^{1}\left(x,y\right),\cdots ,I^{12}\left(x,y\right)\right)}{I_{ref}\left(x,y\right)}.$
(2) $I_{dev}\left(x,y\right)$ is low-pass filtered with a Gaussian filter with the kernel size $ks_{2}$. The resulting image is called $I_{dev,\;\;smoothed\;}\left(x,y\right)$.
(3) (a) For voxels with $I_{dev,\;\;smoothed\;}\left(x,y\right)>thr$, the repetition with the lowest signal is excluded from the signal calculation for this voxel. Note that the excluded signals may originate from different repetitions in adjacent voxels (i.e., not a complete image is excluded, although this is suggested differently in the figure).
(4) (b, first iteration only) For voxels with $I_{dev,\;\;smoothed\;}\left(x,y\right)>thr$, the relative standard deviation after (i) exclusion of the minimal signal and after (ii) exclusion of the maximal signal is compared. If the standard deviation is stronger reduced in case (ii), the highest signal is excluded in addition. This step is important to prevent the exclusion of many low signals in cases where the high standard deviation occurs due to one accidentally high signal.
(5) A new image $I_{new}\left(x,y\right)$ is calculated with the remaining signals per voxel.
(6) A new standard deviation map $I_{dev}\left(x,y\right)$ is calculated as the ratio of the standard deviation map of the remaining images and $I_{new}\left(x,y\right)$. $I_{dev}\left(x,y\right)\;=\frac{\mathrm{std}\left(I^{1}\left(x,y\right),\cdots ,I^{11}\left(x,y\right)\right)}{I_{new}\left(x,y\right)}$

If k>1, the process continues at step (2). Steps (2) to (5) are performed k times. After the last iteration, the current $I_{new}\left(x,y\right)$ is used as the resulting image.

## Appendix B Supplementary material

Supplementary data 1 Detailed optimization report of the five conventional algorithms.
