# Supplementary material for: Feature-guided deep learning reduces signal loss and increases lesion CNR in diffusion-weighted imaging of the liver
Source: Z Med Phys. 2023 Aug 4;34(2):258–69. doi: 10.1016/j.zemedi.2023.07.005 (PMC11156785; doi:10.1016/j.zemedi.2023.07.005)
Supplement: Supplementary data 1 — Detailed optimization report of the five conventional algorithms. [file mmc1.pdf]

1. Description of the algorithms

For all evaluations, the magnitude images were used. The algorithms had also been used in a previous study to evaluate the flow-compensated data[1]. A visualization can be found in the earlier study[1].

Algorithm 1: Weighted averaging

Gaussian-filtered versions of the original images were used as weight maps w_{dm}, where the index d denotes the diffusion direction and m, the repetition:

$$w_{dm} = \text{Gauss}_{ks_1 \times ks_1} * I_{dm}$$

The asterisk denotes the discrete convolution operator in the image plane, and ks_1 is the size of the filter kernel.

Inspired by Ichikawa et al.[2], the final image was then calculated as follows:

$$I_{\text{weighted averaging}} = \sqrt[3]{\prod_{d=x,y,z} \frac{\sum_m w_{dm}^n I_{dm}}{\sum_m w_{dm}^n}}$$

where n is the exponent of the weight maps. The kernel size ks_1 was sampled in steps of $2\ \text{voxels}\ (= 3.125\ \text{mm})$ from $1\ \text{voxel}$ (no filter applied) to $49\ \text{voxels}\ (= 7.7\ \text{cm})$; n was sampled in steps of 1 from 1 to 10.

Algorithm 2: p-mean algorithm

In a similar manner to the method proposed by Liau et al.[3], the final image was calculated as follows:

$$I_{\text{pmean}} = \sqrt[3]{\prod_{d=x,y,z} \sqrt[p]{\frac{\sum_m I_{dm}^p}{4}}}$$

The parameter p can assume any positive value. This calculation was performed separately for each voxel. p was sampled in steps of 0.1 between $p = 1$ and $p = 20$.

Algorithm 3: Percentile algorithm

The final image was calculated as the q-th percentile of the single images:

$$I_{\text{new}} = \text{perc}_{\text{q}}(I_{x1}, I_{x2}, I_{x3}, I_{x4}, I_{y1}, I_{y2}, I_{y3}, I_{y4}, I_{z1}, I_{z2}, I_{z3}, I_{z4})$$

This calculation was performed separately for each voxel. q was sampled in steps of 5 from 0 to 100.

Algorithm 4: Outlier exclusion algorithm

See Appendix of main article.

Algorithm 5: Exception set algorithm (by Arning et al.[4])

This algorithm finds the subset I_j (called "exception set") of the 12 elements I, whose exclusion maximizes the homogeneity of the remaining set while not excluding more elements than necessary.

All $\sum_{i=0}^{maxout} \binom{12}{i}$ possible signal subsets I_j with up to $maxout$ elements are calculated. For each subset, a smoothing factor SF is calculated:

$$SF(I_j) = |I \backslash I_j| \cdot \left(Var(I) - Var(I \backslash I_j)\right),$$

where $|\cdot|$ denotes the number of elements in the set and $Var(\cdot)$ denotes the variance of the elements in the set.

For each voxel, the set I_j with the highest SF was excluded. The new image I_{new} was calculated with a weighted geometric mean.

$maxout$ was sampled from 1 to 12.

2. Optimization and results

Identical window/level settings were used for all images compared directly.

Weighted averaging

In Figure 1A, the panel on the left shows how Q_{total} varies in relation to kernel size ks_1 and exponent n. The highest Q_{total} of 1.087 was achieved for $ks_1 =$ 15 voxels (= 2.3 cm) and an exponent n of 3. For these values, the image quality according to Q_{total} changed significantly (P < 0.0001). In the two other plots shown in Figure 1A, one parameter is fixed to these optimal values (i.e., to ks_1 = 15 voxels or $n = 3$) and the relationship between Q_{total} and the subscores for the other parameter is plotted. In Figures 1B and C, representative images, postprocessed with different parameters of ks_1 and n, are shown. The larger kernel size $ks_1 = 15$ voxels results in a clearer depiction of vessels than the small kernel size $ks_1 = 1$ voxel (Figure 1B, black arrow heads). Compared to the reference image, $n = 3$ leads to a higher lesion conspicuity (Figure 1C, white arrow).

In summary, Q_{total} constrains the free parameters of the weighted averaging algorithm as follows:

- $ks_1 \approx 15$ voxels, which corresponds to 2.3 cm
- $2 \leq n \leq 4$, best $n \approx 3$

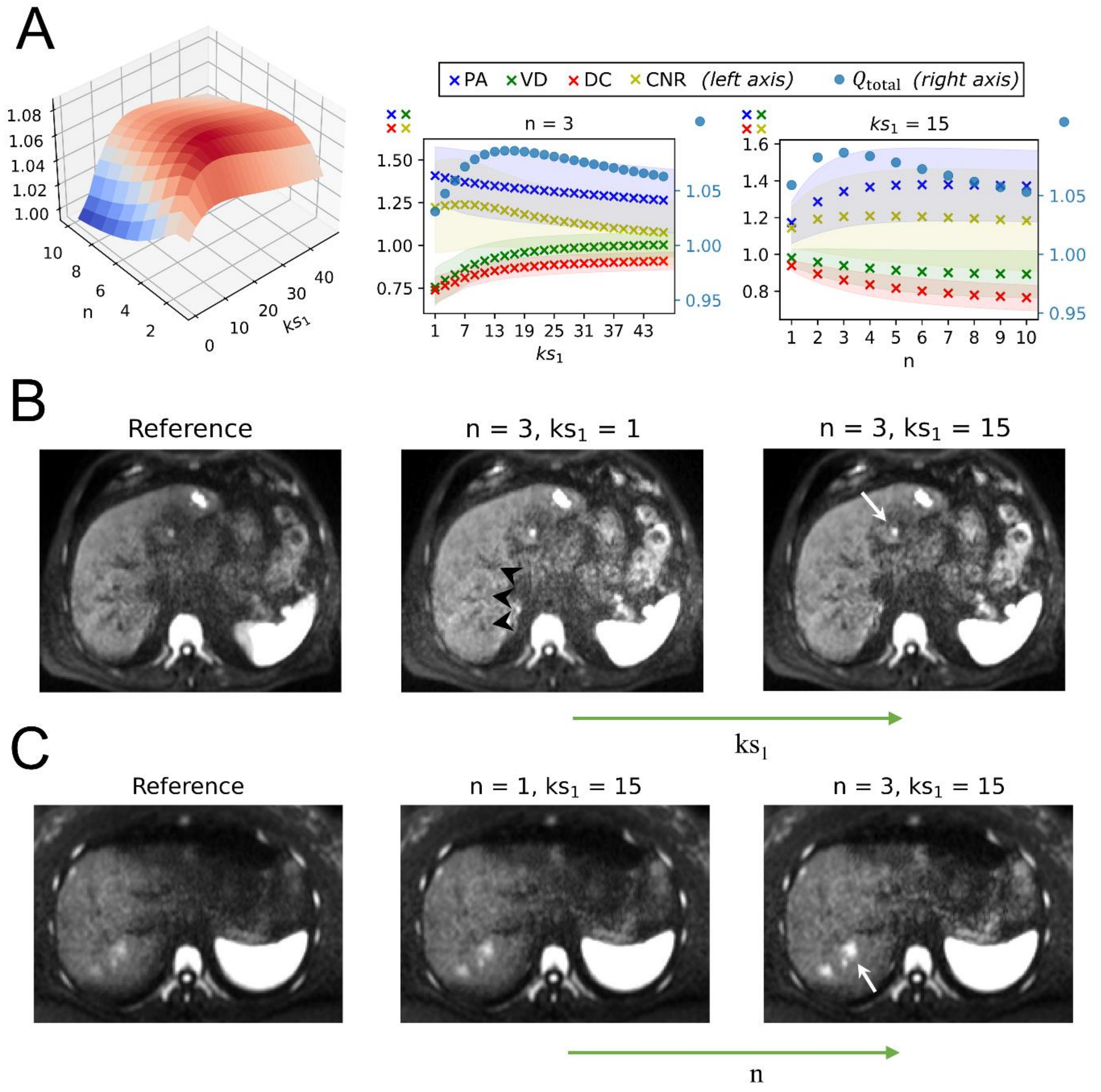

Figure 1: Weighted averaging algorithm. (A) Left plot: variation of Q_{total} with respect to n and ks_1. Center and right plots: the relationship between Q_{total} and the subscores for one parameter, while the other parameter remains fixed to its optimum. The right y-axes represent Q_{total} and are scaled differently. Standard deviations of the subscores are shown as shaded regions. (B) Images at different values of ks_1. The black arrowheads mark vessels that are not clearly visible, and the arrow marks a lesion with increased conspicuity at $n = 3$. (C) Images at different values of n. The white arrow marks a lesion with increased conspicuity at $n = 3$. PA, pulsation artifact; VD, vessel darkness; DC, data consistency; CNR, contrast-to-noise ratio; Q_{total}, total quality score.

p-mean algorithm

Figure 2 shows how the different subscores and Q_{total} vary as a function of p for the p-mean algorithm. The highest Q_{total} of 1.036 was achieved for $p = 4.0$. For this value, the image quality according to Q_{total} changed significantly (P = 0.0004). Additionally, representative images are shown. At $p = 4$, a lesion in the left liver lobe was more clearly visible than in the reference image (white arrow). At $p = 20$, the vessels are more difficult to discern and the image appears noisier.

In summary, the Q_{total} constrains the free parameter p as follows:

- $3 \leq p \leq 5$, best $p \approx 4.0$

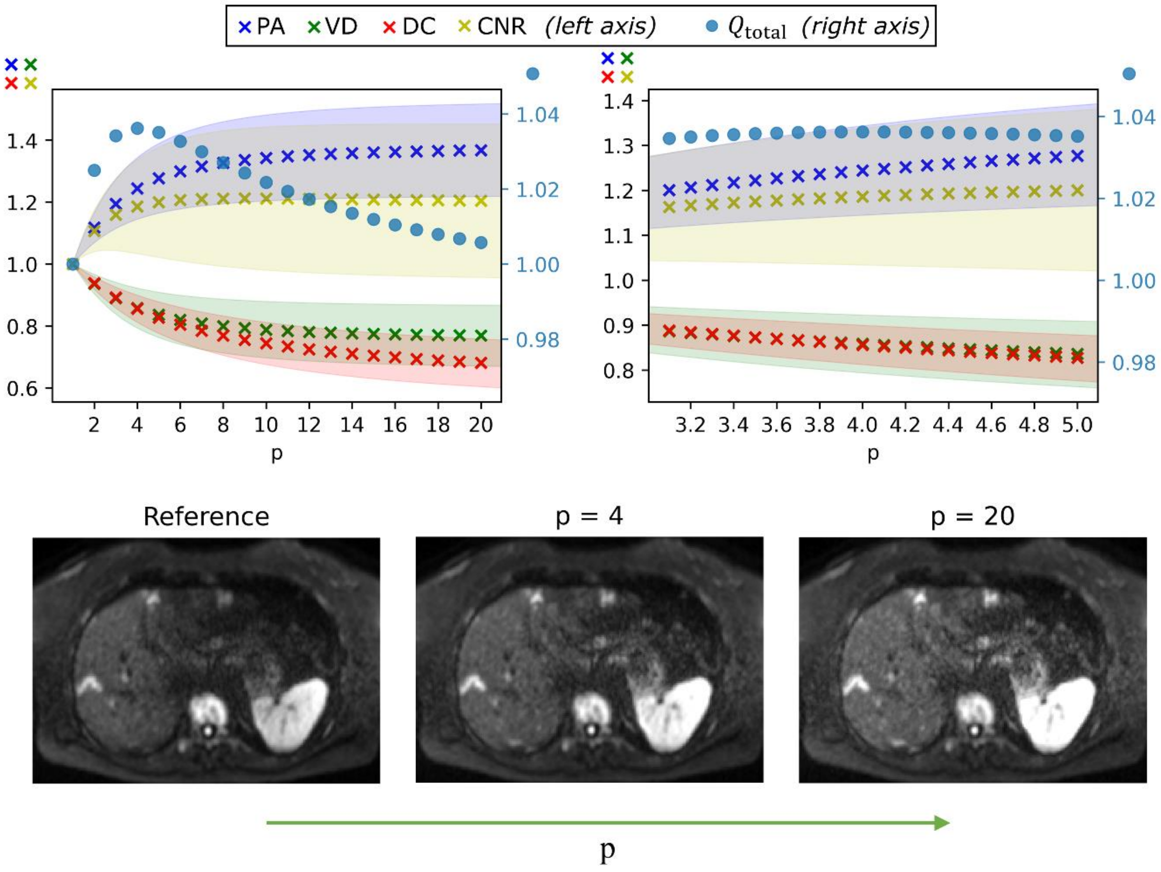

Figure 2: p-mean algorithm. (Top row) The variation of Q_{total} and the subscores with respect to p. In the left plot, only integer values of p are shown, while the right plot shows all values between $p = 3$ and $p = 5$. The right y-axes represent Q_{total} and are scaled differently. Standard deviations of the subscores are shown as shaded regions. (Bottom row) Images at different values of p. PA, pulsation artifact; VD, vessel darkness; DC, data consistency; CNR, contrast-to-noise ratio; Q_{total}, total quality score.

Percentile algorithm

Figure 3 shows the results for the percentile algorithm for different values of percentile q. The highest Q_{total} of 1.004 was reached at $q = 85$. The image quality according to Q_{total} did not change significantly (P = 0.856). The representative image for $q = 0$ is rather dark and of generally low quality, and the lesions are hardly visible. The image for $q = 50$ is of much better quality and the lesions are clearly visible. For $q = 85$, the left liver lobe is more clearly visible than for $q = 50$ or the reference image, but the image appears noisier and the vessels are less dark. These trends continue and become more pronounced for $q = 100$.

In summary, the percentile algorithm does not increase the image quality significantly, but if it is used, Q_{total} constrains the free parameter q as follows:

- $70 \le q \le 90$, best $q \approx 85$

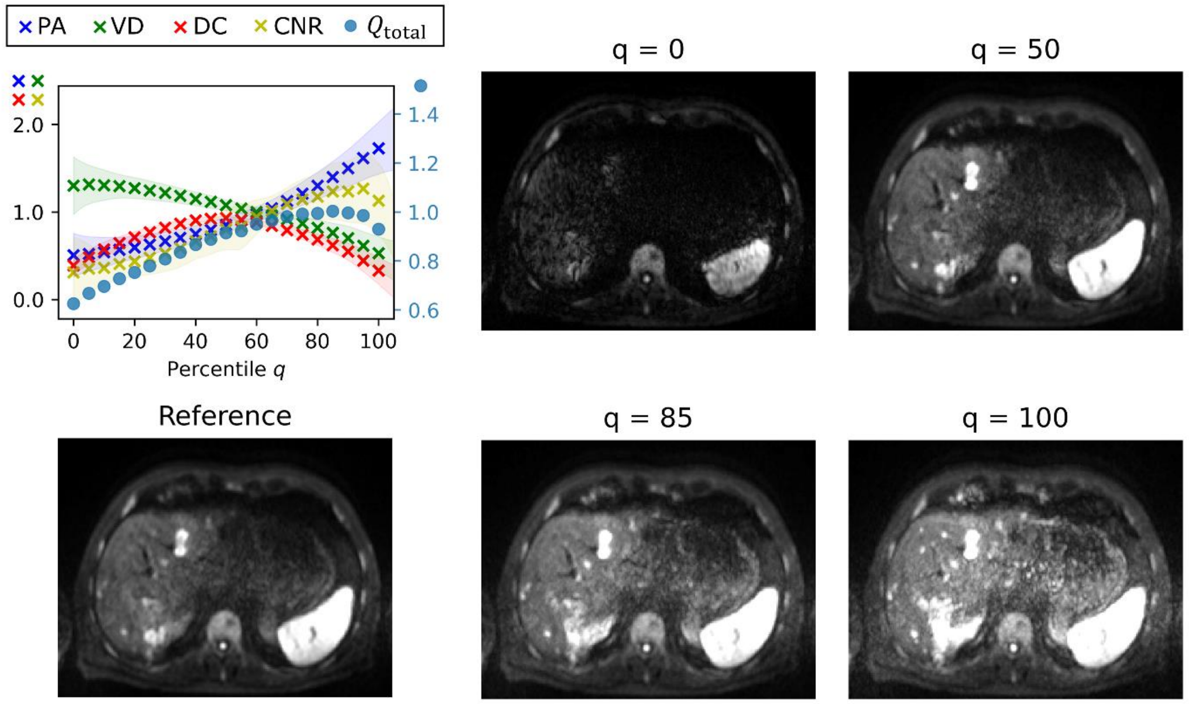

Figure 3: Percentile algorithm. (Top left) Variation of Q_{total} and subscores with respect to q. The right y-axis represents Q_{total} and is scaled differently. Standard deviations of the subscores are shown as shaded regions. (Surrounding panels) Images at different values of q. PA, pulsation artifact; VD, vessel darkness; DC, data consistency; CNR, contrast-to-noise ratio; Q_{total}, total quality score.

Outlier exclusion algorithm

Figure 4 shows the results for the outlier exclusion algorithm. The relationships between Q_{total} and two of the three free parameters are shown in Figure 4A. The third free parameter is fixed to the value at which Q_{total} becomes maximal. The highest Q_{total} value of 1.115 was achieved for the kernel size $ks_2 = 21$ voxels (= 3.3 cm), the correction threshold $thr = 0.3$, and the number of iterations $k = 10$ (i.e., the optimal value of k lies at the boundary of the investigated values). For these values, the image quality according to Q_{total} changed significantly ($P < 0.0001$)

Figure 4B shows how the subscores and Q_{total} vary with respect to a single parameter, with the other two parameters fixed to their optimal values.

Representative images, with two parameters fixed and one varied, are shown in Figure 4C-E. In the example images shown in Fig 4C, increasing the number of iterations k increases the brightness in the left liver lobe. The negative effects of larger k values, such as slightly brighter vessels, are minor, however. Figure 4D shows example images in which artefactual small-size hyperintensities are reduced by the larger kernel size ks_2. Figure 4E shows another example image, in which a reduction in vessel darkness is countered by increasing the threshold thr, which also reduces the perceived noise in the liver.

In summary, Q_{total} constrains the free parameters of the outlier exclusion algorithm as follows:

- $thr \approx 0.3$
- $k \geq 9$, which corresponds to the exclusion of up to 9 out of the 12, i.e., approximately 75 % of the available data
- $ks_2 \approx$ 15–30 voxels, which corresponds to 2.3–4.7 cm

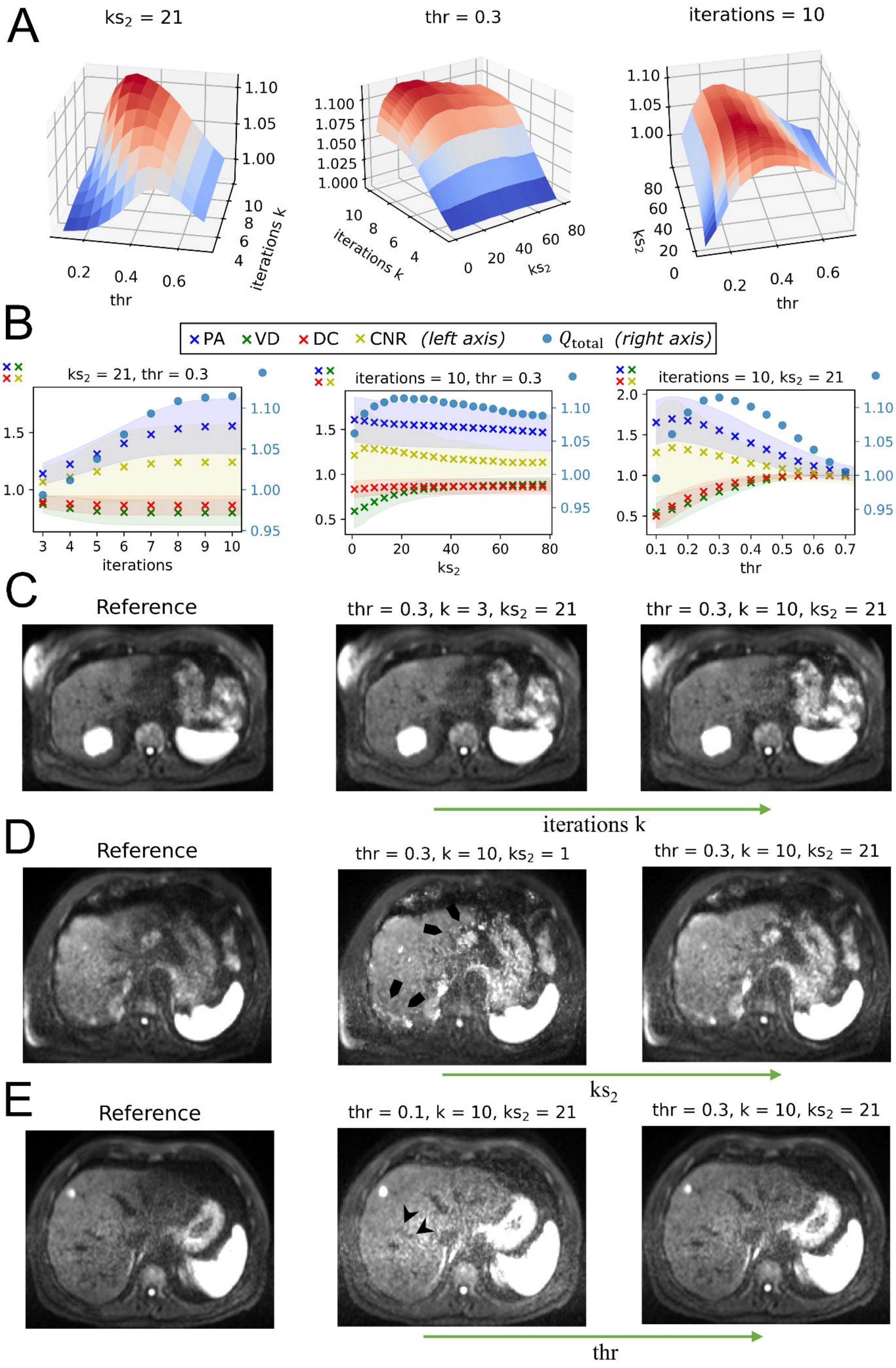
A
ks_2 = 21
thr = 0.3
iterations = 10
thr
iterations k
ks_2
B
PA VD DC CNR (left axis) Q_{total} (right axis)
ks_2 = 21, thr = 0.3
iterations = 10, thr = 0.3
iterations = 10, ks_2 = 21
iterations
ks_2
thr
C
Reference
thr = 0.3, k = 3, ks_2 = 21
thr = 0.3, k = 10, ks_2 = 21
iterations k
D
Reference
thr = 0.3, k = 10, ks_2 = 1
thr = 0.3, k = 10, ks_2 = 21
ks_2
E
Reference
thr = 0.1, k = 10, ks_2 = 21
thr = 0.3, k = 10, ks_2 = 21
thr

Figure 4: Outlier exclusion algorithm. (A) Variation of Q_{total} with respect to two parameters, while the third parameter is fixed. (B) Variation of Q_{total} and the subscores with respect to one parameter, while the other parameters are fixed to their optimal values. The right y-axes refer to Q_{total} and are scaled differently. Standard deviations of the subscores are shown as shaded regions. (C) Images at different values of k. (D) Images at different values of ks_2. The thick black arrows mark artifactual regions. (E) Images at different values of thr. The black arrowheads mark a vessel that is not clearly visible.

Exception set algorithm

Figure 5 shows the Q_{total} and the subscores for the exception set algorithm. Q_{total} was lower than 1, indicating a decreased image quality. The highest score of 0.936 was achieved for $maxout = 1$. At $maxout = 1$, the image quality according to Q_{total} changed significantly ($P < 0.0001$). The representative images in Figure S1 indicate a worsening of the pulsation artifact for all $maxout$ values in comparison to the reference image.

According to the Q_{total} values, the exception set algorithm decreases the image quality. If it is used, the $maxout$ value should be set as follows:

- $maxout = 1$

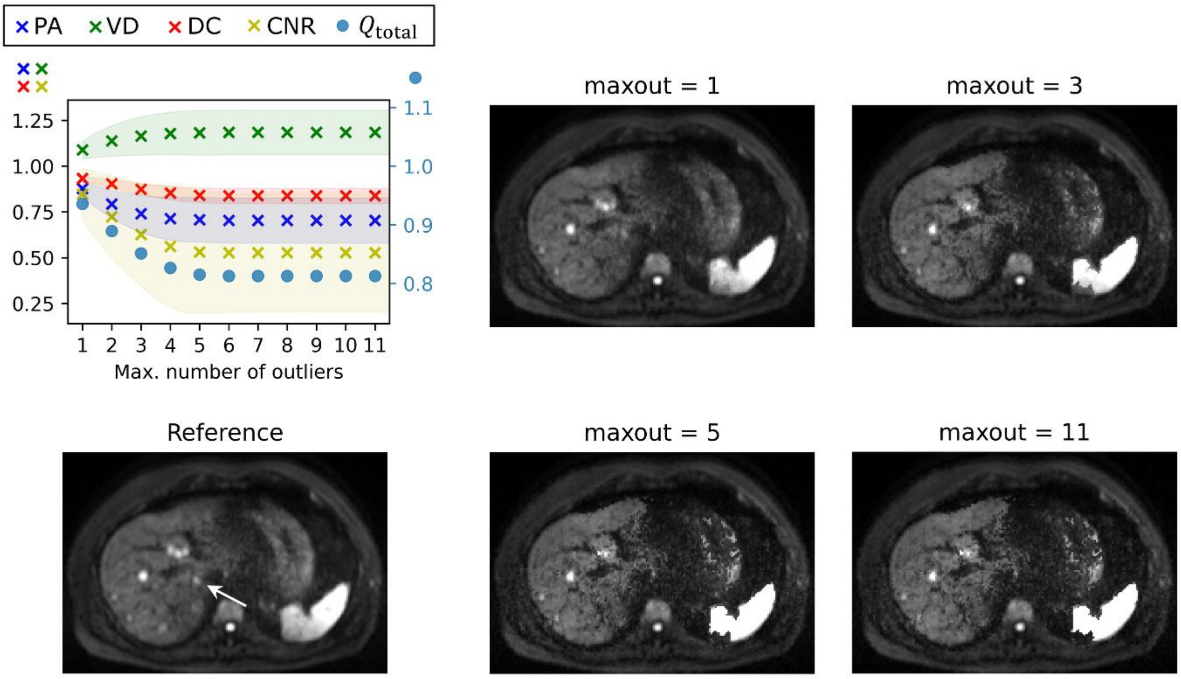

Figure 5: Exception set algorithm. The plot in the top left shows the variation in Q_{total} and subscores with respect to $maxout$. The right y-axis represents Q_{total} and is scaled differently. Standard deviations of the subscores are shown as shaded regions. The surrounding panels show images at different values of $maxout$. The arrow marks a lesion that is no longer visible at high $maxout$ values. PA, pulsation artifact; VD, vessel darkness; DC, data consistency; CNR, contrast-to-noise ratio; Q_{total}, total quality score.

Comparing the optimized algorithms

The total quality scores and subscores obtained for each algorithm with its optimized parameter set are summarized in Table 1. Three of the five analyzed algorithms significantly increased the image quality, according to the Q_{total} values. The outlier exclusion algorithm achieved the highest total quality score ($Q_{\text{total}} = 1.115$), followed by weighted averaging ($Q_{\text{total}} = 1.087$) and p-mean ($Q_{\text{total}} = 1.036$). The percentile algorithm ($Q_{\text{total}} = 1.004$) and the exception set algorithm ($Q_{\text{total}} = 0.936$) did not increase the image quality significantly.

Outlier exclusion performed well in most subcategories (rank 1, 1, and 2 in the subscores PA, CNR, and DC, respectively), but is ranked only 4th for the VD subscore. The algorithm with the second highest Q_{total}, weighted averaging, ranked less well in the subcategories (rank 3, 2, 3, and 3 for the subscores PA, VD, CNR, and DC). The exception set algorithm has the highest subscore in two categories (VD and DC), but also the lowest Q_{total}.

Additional representative images are shown in Figure 6. The weighted averaging, p-mean, percentile, and outlier exclusion algorithms reduced the pulsation artifact and increased lesion visibility, even though the percentile algorithm increased the level of noise and decreased vessel visibility (third column). The exception set algorithm worsened the pulsation artifact and decreased lesion visibility.

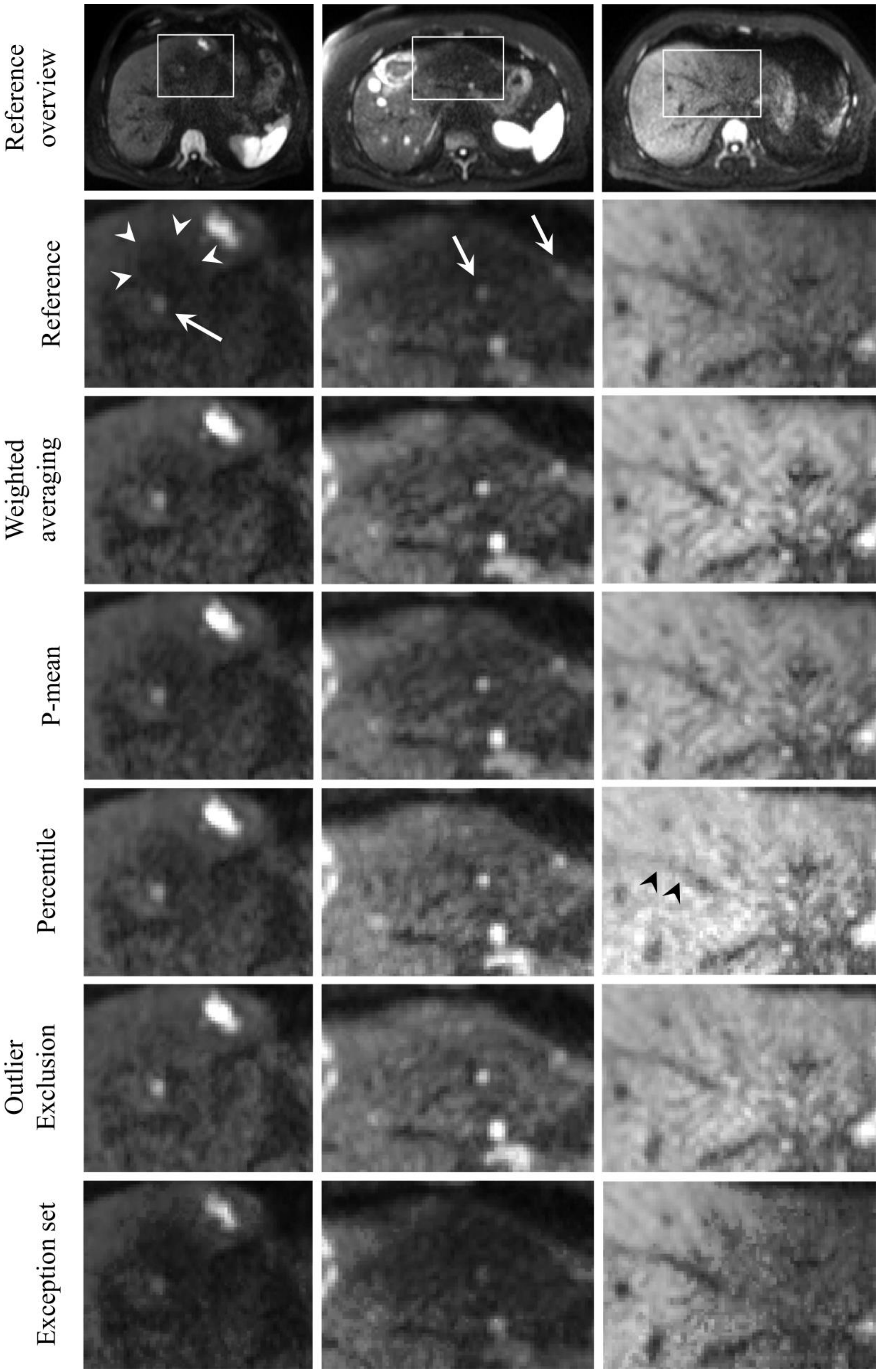

Figure 6: Representative reference images and postprocessed images, which were edited using

the optimized algorithms. (First column) The arrow marks a lesion with changing conspicuity, and the arrowheads indicate a dark region which is best corrected by the outlier exclusion algorithm. (Second column) The arrows mark lesions that are not clearly visible in the reference image. (Third column) The arrowheads in the fifth row indicate a vessel that is not clearly visible.

	PA subscore	VD subscore	CNR subscore<br>- *Left*<br>- *Right*	DC subscore	Q_{total}	*P*-value (Q_{total} different from 1)
Weighted averaging	1.342 ± 0.194	0.939 ± 0.090	1.206 ± 0.215<br>- 1.267 ± 0.371<br>- 1.144 ± 0.218	0.860 ± 0.056	1.087 ± 0.077	< 0.0001
p-mean	1.244 ± 0.099	0.858 ± 0.064	1.186 ± 0.152<br>- 1.223 ± 0.247<br>- 1.150 ± 0.178	0.856 ± 0.044	1.036 ± 0.049	0.0004
Percentile	1.400 ± 0.180	0.762 ± 0.112	1.234 ± 0.320<br>- 1.369 ± 0.604<br>- 1.100 ± 0.214	0.618 ± 0.081	1.004 ± 0.098	0.856
Outlier exclusion	1.556 ± 0.243	0.799 ± 0.110	1.241 ± 0.325<br>- 1.375 ± 0.631<br>- 1.106 ± 0.153	0.862 ± 0.085	1.115 ± 0.107	< 0.0001
Exception set	0.877 ± 0.045	1.088 ± 0.048	0.846 ± 0.139<br>- 0.808 ± 0.226<br>- 0.888 ± 0.162	0.932 ± 0.012	0.936 ± 0.039	< 0.0001

Table 1: Q_{total}, subscores, and p-values for the optimized algorithms. PA, pulsation artifact; VD, vessel darkness; CNR, contrast-to-noise ratio; DC, data consistency; Q_{total}, total quality score.

References

1. Führes T, Saake M, Lorenz J, et al. Reduction of the cardiac pulsation artifact and improvement of lesion conspicuity in flow-compensated diffusion images in the liver-A quantitative evaluation of postprocessing algorithms. *Magn Reson Med.* 2022.
2. Ichikawa S, Motosugi U, Tamada D, et al. Improving the Quality of Diffusion-weighted Imaging of the Left Hepatic Lobe Using Weighted Averaging of Signals from Multiple Excitations. *Magn Reson Med Sci.* 2019;18(3):225-232.
3. Liau J, Lee J, Schroeder ME, Sirlin CB, Bydder M. Cardiac motion in diffusion-weighted MRI of the liver: artifact and a method of correction. *J Magn Reson Imaging.* 2012;35(2):318-327.
4. Arning A, Agrawal R, Raghavan P. A Linear Method for Deviation Detection in Large Databases. Presented at: KDD1996.
